# Evaluation of Chemical Compositions, Antioxidant Capacity and Intracellular Antioxidant Action in Fish Bone Fermented with *Monascus purpureus*

**DOI:** 10.3390/molecules26175288

**Published:** 2021-08-31

**Authors:** Ya-Ting Chen, Shu-Ling Hsieh, Wei-Siang Gao, Li-Jung Yin, Cheng-Di Dong, Chiu-Wen Chen, Reeta-Rani Singhania, Shuchen Hsieh, Shu-Jen Chen

**Affiliations:** 1 Department of Seafood Science, National Kaohsiung University of Science and Technology, Kaohsiung 81157, Taiwan; melodyyu.chen@gmail.com (Y.-T.C.); slhsieh@nkust.edu.tw (S.-L.H.); siangsiang1003@gmail.com (W.-S.G.); ljyin@mail2000.com.tw (L.-J.Y.); 2 Department of Marine Environmental Engineering, National Kaohsiung University of Science and Technology, Kaohsiung 81157, Taiwan; cddong@nkust.edu.tw (C.-D.D.); cwchen@nkust.edu.tw (C.-W.C.); reetasinghania@nkust.edu.tw (R.-R.S.); 3 Department of Chemistry, National Sun Yat-sen University, Kaohsiung 80424, Taiwan; shsieh@faculty.nsysu.edu.tw; 4 Department of Chemical and Materials Engineering, National Kaohsiung University of Science and Technology, Kaohsiung 80778, Taiwan

**Keywords:** fish bone, *Monascus purpureus*, antioxidant, Clone-9 cells

## Abstract

Fish bones (FBs) are aquatic by-products that are sources of antioxidant-active peptides, calcium dietary supplements, and biomedical materials. Usually, fermentation of these by-products via microorganisms brings desirable changes, enhancing their value. This study investigates the value addition of FB when fermented with *Monascus purpureus* (MP) for different time intervals, such as 3 days (F3) and 6 days (F6). The results indicate that the soluble protein, peptide, amino acid and total phenol content, as well as the antioxidant capacity (DPPH, ABTS^+^ radical scavenging activity, and relative reducing power), of F3 and F6 were significantly increased after fermentation. Furthermore, the ROS contents of F3 and F6 were reduced to a greater extent than that of hydrogen peroxide (H_2_O_2_) in Clone-9 cells. The MMP integrity, as well as the SOD, CAT, and GPx activity, of F3 and F6 were also increased significantly compared to the H_2_O_2_ in Clone-9 cells. Notably, F3 and F6 displayed significant reductions in ROS content, as well as elevate, SOD activity and MMP integrity in Clone-9 cells, when compared with the native FB. These results indicate that the FBs fermented with MP for 3 days (F3), and 6 days (F6) have antioxidant capacity, with possible applications as natural food supplements.

## 1. Introduction

Fermentation is a metabolic process that brings about chemical changes in organic substrates through enzymatic actions. It is also a natural decomposition process whereby complex organic substances are broken down into simpler compounds by the action of microorganisms, resulting in the production of bioactive compounds with specific nutritional and health functionalities [1]. Recently, the fermentation of aquatic by-products has been attracting much more interest, resulting in the production of bioactive peptides and aromatic compounds, and also enhancing the biofunctional activity of bioactive compounds [2].

*Monascus* is a type of fungi that belongs to the phylum Ascomycota and the family Monascaceae [3]. *Monascus* and its metabolites exhibit various biological activities, including antioxidant, anti-inflammatory, antidiabetic, antiobesity, antihypertensive, and anticancer [4,5]. Previous studies indicate that fermentation via *Monascus* can enhance the biological activity of food and waste. Food fermented with *Monascus*, such as Radix Puerariae, can increase 1,1-diphenyl-2-picrylhydrazyl (DPPH) radical scavenging activity [6], and *Saccharina japonica* can increase 2,2’-azinobis (3-ethylbenzothiazoline-6-sulfonic acid) (ABTS^+^) radical scavenging activity [7]. Fermenting waste with *Monascus,* such as Kinmen sorghum liquor waste, can increase ABTS^+^ radical scavenging activity [8], and fermenting rice bran with *Monascus* can increase ABTS^+^ and DPPH radical scavenging activity, iron-chelating activity, and reducing power [9]. However, none of the research to date has carried out the fermentation of fish bone with *Monascus* for value addition.

Over 22.1 million tons of aquatic by-products are produced every year worldwide, such as bone, frames, fins, heads, skins, scales and viscera [10,11]. In order to utilize the aquatic by-products, it is a better option to convert wastes into new products (including fishmeal) with high added value. Previous studies found that aquatic by-products, such as fish sauce, skin and heads, can be fermented with microorganisms to obtain bioactive peptides with an antioxidant capacity [2]. Fish bone contains nutrients, such as protein and phosphorus. Research indicates that striped catfish bones [12] and salmon bones [13] are hydrolyzed by proteases to obtain hydrolysates with an antioxidant capacity. These studies indicate that fish bones have antioxidant potential. However, there is currently no relevant research on the antioxidant capacity of fermented fish bones.

The mitochondria are a crucial organelle for cellular activity because they generate ATP. However, harmful reactive oxygen species (ROS) are produced by mitochondrial electron transport, necessitating the activation of antioxidative defense to preserve homeostatic mitochondrial function and mitochondrial membrane potential (MMP) integrity [14]. ROS are highly reactive molecules that can damage nucleic acids, lipids and proteins in cells, causing oxidative stress, loss of cell function, and finally disease or cell death [15,16]. Thus, scavenging ROS is important for the protection of living organisms. Antioxidants, including the enzymes catalase (CAT), superoxide dismutase (SOD) and glutathione peroxidase (GPx), are powerful ROS scavengers that protect cells.

The objective of this study was to investigate the chemical composition, antioxidant capacity, and intracellular antioxidant action of fish bone (FB) fermented with *Monascus purpureus* (MP) for different time intervals, such as 3 days (F3) and 6 days (F6). The chemical composition (soluble protein, peptide, amino acid, and total phenolic contents), antioxidant capacity (DPPH, ABTS^+^ radical scavenging activity, and relative reducing power), and antioxidant action of hydrogen peroxide (H_2_O_2_)-induced Clone-9 cells (ROS content, mitochondrial membrane potential (MMP) integrity, and the activity of SOD, CAT and GPx) were evaluated to determine the potential of FB fermented with MP for its antioxidant capacity.

## 2. Results

### 2.1. Chemical Composition of FB, F3, and F6

The chemical composition is given in Table 1. These results show that the soluble protein and peptide contents of F3 and F6 were significantly higher than those of FB. The total phenolic content of F3 and F6 was approximately increased by 4.2- and 1.2-fold compared to FB, respectively.

The free amino acid composition is given in Table 2. The total free amino acid contents of F3 (88.44 mg/100 mL) and F6 (54.66 mg/100 mL) were significantly higher than those of FB (26.76 mg/100 mL). Furthermore, the amino acid composition analysis shows that the essential amino acid as well as non-essential amino acid contents of F3 and F6 were significantly higher than those of FB. Specifically, we found that essential amino acids, such as histidine, isoleucine, leucine, methionine and valine, and non-essential amino acids such as alanine, glutamic acid, glycine and serine, were significantly increased in F3 compared to FB. The contents of essential amino acids, such as histidine, phenylalanine, threonine and tryptophan, and non-essential amino acids, namely, arginine, asparagine, aspartic acid, glutamine, proline and tyrosine, were significantly increased in F6 compared to FB.

### 2.2. Antioxidant Capacity of FB, F3, and F6

**Figure 1:**
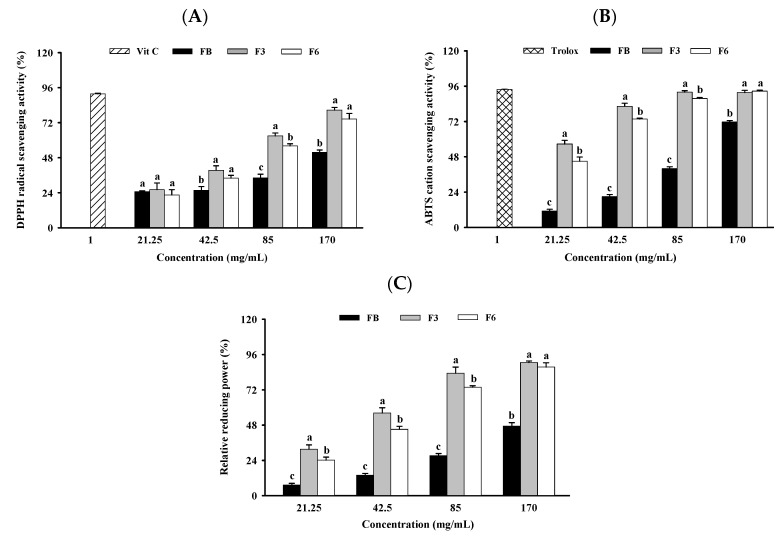
Effect of FB, F3, and F6 on (**A**) DPPH, (**B**) ABTS^+^ radical scavenging activity, and (**C**) relative reducing power. Data are expressed as mean ± SD (*n* = 3). Duncan’s multiple range tests were performed, and different superscripts (a,b,c) assigned to the same concentration indicate significant differences (*p* < 0.05). FB: fish bone; F3: fish bone fermented with *Monascus purpureus* for 3 days; F6: fish bone fermented with *Monascus purpureus* for 6 days.

The antioxidant capacity of DPPH, the ABTS^+^ radical scavenging ability, and the relative reducing power were determined by spectrophotometric methods. The DPPH radical scavenging activities of FB, F3, and F6 with concentrations from 21.25 to 170 mg/mL were 25~52%, 26~81% and 23~75%, respectively (Figure 1A). The ABTS^+^ radical scavenging activities of FB, F3, and F6 in concentrations from 21.25 to 170 mg/mL were 11~72%, 57~92% and 45~93%, respectively (Figure 1B). The relative reducing powers of FB, F3, and F6 in concentrations from 21.25 to 170 mg/mL were 7~47%, 32~91% and 24~88%, respectively (Figure 1C). These results show that the DPPH radical, ABTS^+^ radical scavenging activities and relative reducing powers of F3 and F6 at 42.5, 85 and 170 mg/mL were significantly higher than those of FB.

Furthermore, F3 and F6 at 85 mg/mL exhibited approximately 1.8- and 1.6-fold higher DPPH radical scavenging activities than FB. F3 and F6 at 21.25 mg/mL exhibited approximately 5.0- and 4.0-fold higher ABTS^+^ radical scavenging activities than FB. F3 and F6 at 21.25 mg/mL exhibited approximately 4.4- and 3.3-fold higher relative reducing power than FB.

### 2.3. Cell Viability of FB, F3, and F6

**Figure 2:**
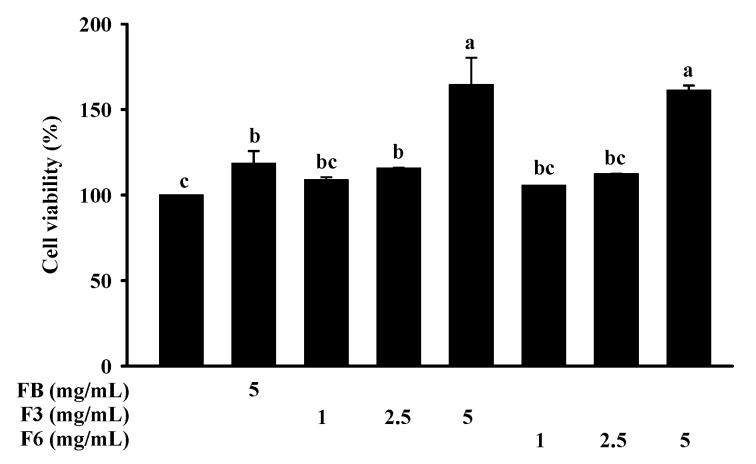
Effect of FB, F3, and F6 on cell viability in Clone-9 cells. Data are expressed as mean ± SD (*n* = 3). Tukey’s tests were performed, with similar superscripts among each group (a,b,c) indicating non-significant difference (*p* < 0.05). FB: fish bone; F3: fish bone fermented with *Monascus purpureus* for 3 days; F6: fish bone fermented with *Monascus purpureus* for 6 days.

The cell viability of FB, F3, and F6 on Clone-9 cells was determined via 3-(4,5-dimethyl-2-yl)-2,5-diphenyltetrazolium bromide (MTT) assays. The viabilities of the Clone-9 cells treated with FB (5 mg/mL), F3 (1, 2.5, 5 mg/mL), and F6 (1, 2.5, 5 mg/mL) for 24 h were not significantly different compared to those of the control cells (Figure 2).

### 2.4. Intracellular Antioxidant Action of FB, F3, and F6

Intracellular antioxidant action, including ROS content, mitochondrial membrane potential (MMP) integrity and SOD, CAT and GPx activity, was determined in H_2_O_2_-induced Clone-9 cells.

**Figure 3:**
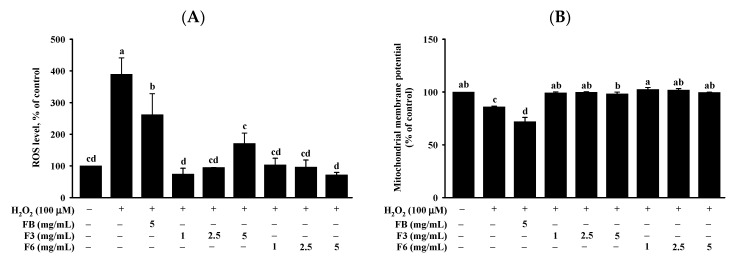
Effect of FB, F3, and F6 on (**A**) ROS content and (**B**) MMP integrity in H_2_O_2_-induced Clone-9 cells. Data are expressed as mean ± SD (*n* = 3). Tukey’s tests were performed, with similar superscripts (a–d) among each group indicating non-significant difference (*p* < 0.05). FB: fish bone; F3: fish bone fermented with *Monascus purpureus* for 3 days; F6: fish bone fermented with *Monascus purpureus* for 6 days.

The production of ROS was assessed by 2’,7’-dichlorofluorescin diacetate (DCFH-DA) assay. The content of ROS was significantly decreased by the treatment of FB, F3, and F6 in cells compared with H_2_O_2_. The integrity of MMP, assessed by 5,5,6,6-tetrachloro-1, 1,3,3-tetraethyl benzimidazol-carbocyanine iodide (JC-1 dye) assay, is given in Figure 3A. The integrity of MMP was significantly elevated in cells by the F3 (1,2.5,5 mg/mL) and F6 (1,2.5,5 mg/mL) treatments, compared with H_2_O_2_ (Figure 3B). Notably, F3 and F6 showed significant reductions in ROS content, and elevated MMP expressions, in cells compared with FB.

**Figure 4:**
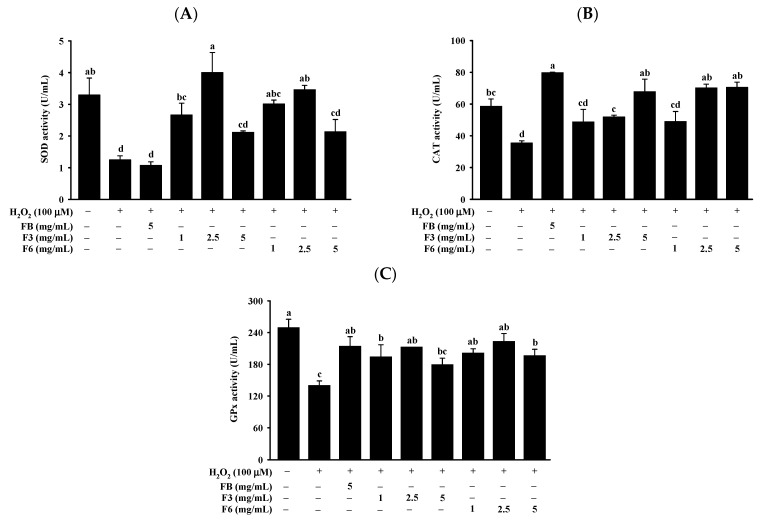
Effect of FB, F3, and F6 on activity of (**A**) SOD, (**B**) CAT, and (**C**) GPx in H_2_O_2_-induced Clone-9 cells. Data are expressed as mean ± SD (*n* = 3). Tukey’s tests were performed, with similar superscripts (a–d) among each group indicating non-significant difference (*p* < 0.05). FB: fish bone; F3: fish bone fermented with *Monascus purpureus* for 3 days; F6: fish bone fermented with *Monascus purpureus* for 6 days.

The enzyme activity of SOD, CAT and GPx was assessed by spectrophotometric methods. The activity of SOD was enhanced in cells by the F3 (1,2.5 mg/mL) and F6 (1,2.5 mg/mL) treatments compared with H_2_O_2_ (Figure 4A). We found that this increase in SOD activity was higher with F3 and F6 than with FB. The activity of CAT was increased in cells by the treatment of FB (5 mg/mL), F3 (2.5,5 mg/mL), and F6 (2.5,5 mg/mL), compared with H_2_O_2_ (Figure 4B). The activity of GPx was significantly increased in cells by the treatment of FB (5 mg/mL), F3 (1,2.5 mg/mL), and F6 (1,2.5,5 mg/mL), compared with H_2_O_2_ (Figure 4C). However, no significant differences in CAT or GPx activity were found in cells between the treatment of FB, F3, and F6.

## 3. Discussion

Previous studies have demonstrated that the fermentation process can increase the antioxidant capacity of biological material [17,18]. A fermented extract of *Lactobacillus sakei* from a mixture of *Undaria pinnatifida, Saccharina japonica,* and *Gloiopeltis furcate* can increase the radical scavenging activity of ABTS^+^ and DPPH [19]. *Cordyceps militaris* mycelia-fermented *Undaria pinnatifida* can increase reducing power and DPPH radical scavenging activity [20]. *Lactobacillus plantarum* fermented alone can increase reducing power and ABTS^+^ and DPPH radical scavenging activities [21]. Moreover, *Bacillus* sp.-fermented cod protein hydrolysate can increase reducing power and DPPH radical scavenging activity [22]. In the present study, the DPPH and ABTS^+^ radical scavenging activities, and relative reducing powers, of F3 and F6 were significantly higher than those in FB. These results show that fermented aquatic organisms can enhance antioxidant capacity by increasing DPPH and ABTS^+^ radical scavenging activity or reducing power.

Vijayabaskar and Shiyamala [23] showed that antioxidant capacity is related to the increase in phenol content. Moreover, research has demonstrated that fermented Radix Puerariae [6], barley seeds [24] and king coconut water [25] can enhance antioxidant capacity and increase total phenolic content. This study found that the total phenolic content of F3 and F6 was approximately increased by 4.2- and 3.3-fold compared to FB, respectively. These results show that the enhanced antioxidant capacity of fermentation products may be caused by the increase in the content of total phenolics after fermentation.

Total phenols have antioxidant functions; however, total phenol content does not necessarily increase as the fermentation time increases [26,27]. The total phenol content of fermented *Psidium guajava* L. leaves at 12 days was significantly more reduced compared to at 8 days [27]. The total phenol content, DPPH and ABTS^+^ radical scavenging activity of fermented pearl millet at 10 days were significantly reduced compared to 8 days [28]. Our results are similar to those of previous studies, as the total phenol content of F6 was found to be lower than that of F3. Moreover, the DPPH and ABTS^+^ radical scavenging and reducing activities of F6 at <85 mg/mL were lower than those of F3. These results show that there is no positive correlation between fermentation time, phenol content and antioxidant capacity.

Studies have shown that the antioxidative properties of an amino acid are more related to its classification, structure, and hydrophobicity [29]. Aromatic amino acids can donate protons to electron-deficient radicals, which improves their radical-scavenging properties. Hydrophobic amino acids can protect against macromolecular oxidation by donating photons to reactive radicals [29,30]. Moreover, hydroxy amino acids can promote metal-chelating capacity [31]. Previous studies have shown that *Bacillus licheniformis OPL-007*-fermented shrimp head waste can enhance antioxidant capacity via increases in its free amino acid contents (phenylalanine, lysine, and methionine) [32]. *Weissella* spp.-fermented salted squid had its antioxidant capacity enhanced by increasing its free amino acid contents (threonine, glutamine, and isoleucine) [33]. *Lactobacillus plantarum*-fermented fish has its antioxidant capacity enhanced via increases in its free amino acid contents (alanine, leucine and proline) [34]. *Bacillus subtilis*-fermented soybean also derives enhanced antioxidant capacity from increases in its free amino acid contents (histidine, proline, tryptophan, phenylalanine and tyrosine) [35]. *Bacillus subtilis* femented wheat germ derives enhanced antioxidant capacity from increases in free amino acid contents (arginine, glutamic acid, glycine, aspartic acid and alanine) [36]. In this study, we saw significant increases in the contents of aromatic amino acids (methionine 8.3-fold and histidine 2.6-fold) and hydrophobic amino acids (isoleucine 7.2-fold, leucine 6.7-fold and valine 6.6-fold) in F3 compared to FB. Moreover, we saw significant increases in the contents of aromatic amino acids (tryptophan 9.4-fold and histidine 2.3-fold), hydrophobic amino acids (phenylalanine 1.6-fold) and hydroxy amino acids (threonine 1.9-fold) in F6 compared to FB. These results show that the enhanced antioxidant capacities of fermentation products may be caused by increases in the contents of these functional amino acids after fermentation. The result show that fish bone contains more lysine and tyrosine, whereas these contents were lower in F3 but increased in F6. We assume that fermentation caused microorganisms to use these amino acids, as nutrients were decreased [37]. However, with increases in fermentation time, these *Monascus purpureus* have also been known to produce amino acids [38].

The total free amino acid content of F6 (54.66 mg/100 mL) is lower than that of F3 (88.44 mg/100 mL). Previous studies have shown that the total free amino acid content of fermented Pu-erh tea at 21~49 days was significantly reduced compared to that at 14 days [39]. The total free amino acid content of salt-fermented shrimp paste at 360 days was significantly reduced compared to that at 90 days [40]. Total free amino acids do not necessarily increase as fermentation time increases, which may be attributed to the consumption of nutrients by microorganisms, or some other complex reactions, such as enzymatic conversion and Maillard reaction during fermentation [39].

The mitochondria are a major locus of ROS generation in cells; this occurs when the mitochondrial membrane potential declines, which disturbs intracellular ATP synthesis and the generation of ROS. Then, ROS is catalytically converted into molecular oxygen and water by CAT, SOD and GPx, consequently curtailing cell and tissue damage (oxidative stress). The efficiency of action of SOD, CAT, and GPx in the overall antioxidant defense strategy is therefore critical [41,42]. Furthermore, H_2_O_2_ is a primary component of the intracellular ROS produced throughout a variety of physiological and pathological activities, and it causes oxidative damage [43]. Previous studies have shown that when cells are subjected to H_2_O_2_, the content of ROS is increased, while the activities of SOD, CAT and GPx are decreased, compared with untreated cells [43,44,45,46]. In the present study, the use of H_2_O_2_ to induce Clone-9 cells has the same result as in previous studies. It indicates that the H_2_O_2_-induced oxidative stress of cells could be caused by increases in the content of ROS and reductions in the activity of SOD, CAT and GPx. However, other enzymes, such as peroxiredoxins, involved in the antioxidant defense system may also induce antioxidant capacity, which we have not confirmed in this study and will be the focus of future studies. The previous report indicated that fermented metabolites, such as gut metabolites of blackberry anthocyanin extract [47] and lovastatin [48], can reduce the content of ROS and maintain the integrity of MMP in cells. Fermented Zijuan Pu-erh tea can increase SOD enzyme activity in ECV340 cells [49]. Sterilized lemon fermented product (LFP) and non-sterilized lemon product (NLFP) can reduce the content of ROS, increase CAT, SOD and GPx enzyme activity, and maintain the integrity of MMP in Clone-9 cells [44]. Fermented black ginseng can increase the activity of CAT, SOD and GPx, and reduce the content of ROS in HepG2 cells [43]. The above studies provide evidence that fermented products can enhance intracellular antioxidant capacity by reducing the content of ROS, maintaining the integrity of MMP and regulating the activity of antioxidant enzymes, especially SOD, CAT and GPx. In this study, FB was shown to reduce the content of ROS, and increase the CAT and GPx activity in Clone-9 cells. However, F3 and F6 protected Clone 9 cells from H_2_O_2_-induced oxidation, reducing the content of ROS by elevating the integrity of MMP and increasing the activity of SOD, CAT and GPx in cells. Notably, F3 and F6 were better than FB at reducing the content of ROS, elevating the integrity of MMP and increasing the activity of SOD. These results suggest that F3 and F6 can protect Clone 9 cells from oxidation induced by H_2_O_2_ more effectively than FB.

## 4. Materials and Methods

### 4.1. Materials

Milkfish (*Chanos chanos*) bones were purchased from a local aquatic product supplier (Kaohsiung, Taiwan). *Monascus purpureus* BCRC 31,499 was obtained from the Bioresources Collection and Research Center, Food Industry Research and Development Institute (Hsinchu, Taiwan). All other chemicals were of analytical grade.

### 4.2. Preparation of Sample

Fish bones were extracted from the fish and refrigeration-transported to the laboratory within 2 h. In the laboratory, the fish bones were divided into three parts. The first 100 g of fish bones was minced using a mixer after being combined with 200 mL of deionized water (1:2 *w/v*). The fish bones were then treated in a high-pressure steam sterilizer for 60 min at 121 °C to deactivate the endogenous enzymes and soften them. The second part was mixed with 14% steamed rice and autoclaved at 121 °C for 15 min. The third part was mixed with 5% *Monascus purpureus* in a 250 mL Erlenmeyer flask and fermented at 37 °C for 3 and 6 days on a shaker at 150 rpm. The samples were then autoclaved for 15 min at 121 °C before being dried in a freeze-drier at −80 °C. These fermented products were codified as F3 and F6. F3 comprises fish bone fermented with *Monascus purpureus* for 3 days, and F6 comprises fish bone fermented with *Monascus purpureus* for 6 days. The percentage yield of F3 and F6 was 82% (*w/w*).

Fish bones (100 g) were minced in a mixer with 200 mL of deionized water (1:2 *w/v*). The samples were then autoclaved for 15 min at 121 °C before being dried in a freeze-drier at −80 °C. These products were codified as FB. The percentage yield of the FB was 85% (*w/w*).

In this study, we used the same conditions to prepare three batches of FB, F3, and F6, respectively, for subsequent experimental analyses. Then, three samples were taken from the same batch of FB, F3, and F6, respectively, and analyzed at the same time (for each experiment).

### 4.3. Chemical Composition Analysis

#### 4.3.1. Determination of Soluble Protein Content

According to the Lowry et al. method, the soluble protein contents of FB, F3, and F6 were assessed [50]. The calibration curve was prepared using bovine serum albumin (Scientific Biotech Corp, Taipei, Taiwan) as the standard. At 660 nm, the optical density (OD) was measured, and the results are indicated in mg/mL.

#### 4.3.2. Determination of Peptides Content

According to the Church et al. method, the peptide contents of FB, F3, and F6 were assessed [51]. The calibration curve obtained from the standard Leu-Gly was converted into the peptide content. At 340 nm, the OD was measured, and the results are indicated in mg/mL.

#### 4.3.3. Composition of Free Amino Acid

Using a commercial TRAQ kit (AB Sciex) and a liquid chromatography–mass spectrometry system, the free amino acid compositions of the relevant amino acids were determined. Before injection, the FB, F3 and F6 were filtered through a 0.22 μm PVDF membrane. Free amino acids were chromatographically separated on the Agilent Poroshell 120 EC-C18 (100 mm × 2.1 mm i.d., 2.7µm) column at 50 °C. At a rate of 0.4 mL/min, a binary gradient of water (mobile phase A) and methanol (mobile phase B) containing 0.1% formic acids was passed through the system. In 20 min, the proportion of mobile phase B was gradually increased from 2% to 90%, followed by a 1 min re-equilibration step. Multiple reaction monitoring (MRM) was used to acquire data from the mass spectrometer (AB Sciex Instruments QTRAP 5500), and data processing was performed with the v.1.5 analyst program (AB Sciex). The mass spectrometer parameters as follows—curtain gas: 20.00 psi; nebulizing gas: 60.00 psi; collision-activated dissociation: medium; heating gas: 60.00 psi; electrospray capillary voltage: 5500.00 V; collision energy: 30.00 V; declustering potential: 30.00 V; entrance potential: 10.00 V.

#### 4.3.4. Determination of Total Phenolic Content

According to the Singleton et al. method, the total phenolic content of FB, F3, and F6 was assessed [52]. Briefly, 50 μL of FB, F3, and F6 were mixed with 200 μL of sodium carbonate (5%) and 50 μL of Folin–Ciocalteu phenol reagent (10%), all from Sigma-Aldrich (St. Louis, MO, USA), then left in the dark for 60 min. The OD was determined at 750 nm, using gallic acid as the standard. The results are expressed in μg/mL.

### 4.4. Antioxidant Capacity Analysis

#### 4.4.1. DPPH Radical Scavenging Activity

According to the Shimada et al. method, the DPPH radical scavenging activity of FB, F3, and F6 was assessed [53]. Briefly, 100 μL of FB, F3, and F6 at 21.25, 42.5, 85 and 170 mg/mL, or vitamin C (1 mg/mL) (J.T. Baker, Center Valley, Penn, USA), was mixed with 400 μL ethanol solution (Taiwan Tobacco & Liquor Corporation, Taipei, Taiwan) and 500 μL of 250 μM DPPH reagent (Sigma-Aldrich, St. Louis, MO, USA), and then placed in the dark for 20 min. Then, the mixture was centrifuged (9560× *g*, 25 °C, 30 s) and the supernatant absorbance was determined at 517 nm. The DPPH radical scavenging activity was calculated as follows:

DPPH radical scavenging activity (%) = [1 − (OD of sample/OD of blank)] × 100%

#### 4.4.2. ABTS^+^ Radical Scavenging Activity

According to the Arnao et al. method, the ABTS^+^ radical scavenging activity of FB, F3, and F6 was assessed [54]. A reaction of 2 mM ABTS solution with 70 mM potassium persulfate produced the ABTS radical cation (ABTS^+^) (Sigma-Aldrich, St. Louis, MO, USA). The mixture was put in the dark for 16 h at room temperature before use. The ABTS^+^ solution was diluted with pH 6.6 PBS (Uni-onward, Taipei, Taiwan) to achieve an absorbance of 0.70~0.83 at 734 nm. The diluted ABTS^+^ solution (990 μL) was added to react with 10 μL of FB, F3, and F6 at 21.25, 42.5, 85 and 170 mg/mL in the dark for 6 min, then the OD was measured at 734 nm. The following formula was used to determine the ABTS^+^ radical scavenging activity:

ABTS^+^ radical scavenging activity (%) = [(OD of blank − OD of sample)/OD of control] × 100%

#### 4.4.3. Relative Reducing Power

According to the Oyaizu et al. method, the relative reducing power of FB, F3, and F6 was determined [55]. Briefly, 100 μL of FB, F3, and F6 at 21.25, 42.5, 85 and 170 mg/mL, or vitamin C (1 mg/mL), was mixed with 150 μL of 1% potassium hexacyanoferrate (III) (Sigma-Aldrich, St. Louis, MO, USA), 150 μL of 0.2 M PBS (pH 6.6) and incubated in a 50 °C water bath for 20 min, then rapidly cooled. Then, 120 μL of 0.1% ferric chloride, 4-hydrate, crystal (FeCl_3_·4H_2_O) (J.T. Baker, Center Valley, PA, USA), 600 μL distilled water and 150 μL of trichloroacetic acid (10%, TCA) (Sigma-Aldrich, St. Louis, MO, USA) were mixed and placed in the dark for 10 min. Then, the OD was measured at 700 nm. The following formula was used to determine the relative reducing power:

Relative reducing power (%) = [(OD of sample − OD of blank)/OD of Vit C] × 100%

### 4.5. Intracellular Antioxidant Action Analysis

#### 4.5.1. Cell Culture and Treatment

The Clone-9 cells were purchased from the Bioresource Collection and Research Center (Hsinchu, Taiwan). Clone-9 cells were cultured in Dulbecco’s modified eagle medium (DMEM), supplemented with 3.7 g/L of sodium bicarbonate, 10% fetal bovine serum, and 1% penicillin/streptomycin, all from Gibco (Grand Island, NY, USA) in an environment with humidified 5% CO_2_ at 37 °C.

In the cell viability experiment, Clone-9 cells in 3 cm culture dish (5 × 10^5^ cells/mL) were treated with control (untreated), F3 (1, 2.5 and 5 mg/mL), F6 (1, 2.5 and 5 mg/mL) or FB (5 mg/mL) for 24 h. In the ROS content, MMP integrity and antioxidant enzymes (SOD, CAT and GPx) assays, cells were treated with control (untreated) and F3 (1, 2.5 and 5 mg/mL), F6 (1, 2.5 and 5 mg/mL) or FB (5 mg/mL) combined with 100 μM H_2_O_2_ (Sigma-Aldrich, St. Louis, MO, USA) for 24 h. Cells treated with 100 μM H_2_O_2_ alone served as a positive control group.

#### 4.5.2. Cell Viability Assay

Cell viability was evaluated using the MTT reduction assay [56]. In total, 1 mL of MTT solution (0.1 mg/mL) (Sigma-Aldrich, St. Louis, MO, USA) was added to a 3 cm culture dish of Clone-9 cells for 3 h (5% CO_2_ at 37 °C). One milliliter of isopropanol (J.T Baker, Center Valley, Penn, USA) was added to solubilize purple formazan crystals and kept in a shaker for 15 min. Then, the mixture was centrifuged (9560× *g*, 4 °C, 10 min) and the supernatant was used to assess absorbance at 570 nm. The cell viability was calculated as follows:

Cell viability (%) = (OD of sample/OD of control) × 100%

#### 4.5.3. ROS Content Assay

The medium was removed from the Clone-9 cells cultured for 24 h, and then washed twice with PBS. The cells were then incubated with 1 mL of DMEM medium containing 10 μM DCFH-DA (Sigma-Aldrich, St. Louis, MO, USA) for 30 min at 5% CO2 and 37 °C. Then, the cells were de-attached by Trypsin-EDTA (Gibco, Grand Island, NY, USA) and rinsed in a medium to gain cell samples. The mixture was then centrifuged (201× *g*, 25 °C, 5 min), and the supernatant was removed and dissolved in 1 mL of PBS. The cell count of 1 × 10^6^ cells/mL of cells was dissolved in 100 μL PBS, then 2 μL of 500 μg/mL Hoechst 33,342 (ChemoMetec A/S, Danmark) was added and incubated at 37 °C in a water bath for 5 min. The sample was centrifuged (201× *g*, 25 °C, 5 min), the supernatant was removed, and the cells were washed three times with 300 μL of PBS, and then the pellet was dissolved in 100 μL of PBS on the third wash. Slide-A2 was infused with 30 L of the sample solution. For the measurement of cell fluorescence intensity, the NucleoCounter^®^ NC-3000^TM^ fluorescent imaging cytometer (Chemometec, Allerod, Denmark) was set to the ROS-DCF test.

#### 4.5.4. MMP Assay

Clone-9 cells were de-attached with trypsin and rinsed in a medium to gain cell samples. They were then centrifuged (201× *g*, 25 °C, 5 min), and the supernatant was removed and dissolved in 480 μL of PBS; 6 μL of 200 μg/mL JC-1 dye was then added (ChemoMetec A/S, Danmark) at 37 °C in dry baths for 30 min. The sample was centrifuged (201× *g*, 25 °C, 5 min), the supernatant was removed, and the cells were washed twice with 300 μL of PBS; the pellet was dissolved in 500 μL of PBS on the second wash, and we then added 300 μL of 1 μg/mL DAPI (ChemoMetec A/S, Danmark). A flow cytometer was used to examine the sample solution (BD Accuri C6) (Becton Dickinson, NJ, USA).

#### 4.5.5. SOD Activity Assay

The SOD activity was determined using a commercial kit (SD125, Randox Laboratories Ltd., Crumlin, Country Antrim, UK). In total, 10 μL of each sample solution or standard was added to a 96-well plate, then 50 μL of xanthine oxidase was added and 200 μL of the substrate was mixed under dark conditions. After a 30 s reaction, the absorbance was measured at 505 nm (A1), and after 3 min, the solution was measured again (A2). The SOD activity was calculated as follows:

Δ(A2–A1)/3 = ΔA/min of the standard or sample

SOD activity (U/mL) = 100 − (ΔA standard or sample/min × 100/ΔA1/min)

#### 4.5.6. CAT Activity Assay

A commercial kit was used to measure the CAT activity (707002, Cayman Chemical, Ann Arbor, MI, USA). In total, 20 μL of each sample solution or standard was then added to the 96-well plate, then 100 μL of 5X assay buffer, 20 μL of H_2_O_2_ and 30 μL of methanol were added, and shaking was continued in the dark for 20 min. Then, 30 μL of potassium hydroxide and purpald (4-amino-3-hydrazino-1,2,4-triazol-5-thiol) was added and kept under shaking conditions in the dark for 10 min. Finally, 10 μL of potassium periodate was added and kept under shaking conditions in the dark for 5 min, and the absorbance was measured at 540 nm. When substituting the absorbance into the standard curve to calculate the formaldehyde concentration, the calculation formula is as follows:

CAT activity (U/mL) = formaldehyde concentration × 0.17/0.02 × 1/20 (min)

#### 4.5.7. GPx Activity Assay

The GPx activity was determined by a commercial kit (RS505, Randox Laboratories Ltd., Crumlin, Country Antrim, UK). In total, 10 μL of each sample solution was added into a 96-well plate, then 100 μL of R1 buffer was added, followed by the addition of 4 μL of cumene hydroperoxide under dark conditions. After a 30 s reaction, the absorbance was measured at 340 nm (A1), and after 3 min, the OD was measured again (A2). The GPx activity was calculated as follows:

GPx activity (U/mL) = Δ(A2 − A1) × 8412

### 4.6. Statistical Analysis

In this study, results were obtained from three independent experiments (*n* = 3) and expressed as mean ± standard deviation. All data and statistical analyses were performed using the SPSS software (SPSS version 12.0, USA). Statistical significance was determined using a one-way analysis of variance (ANOVA) of Duncan’s multiple range tests or Tukey’s tests. A *p*-value less than 0.05 indicated a statistically significant result.

## 5. Conclusions

Our results indicate that F3 and F6 had greater radical scavenging activities than FB alone. Furthermore, F3 and F6 can reduce the ROS content, maintain MMP integrity and increase the activity of antioxidant enzymes SOD, CAT and GPx in Clone-9 cells. This could be attributed to the increase in total phenolic and amino acids contents with the antioxidant capacity of F3 and F6 during fermentation. Therefore, these results indicate that F3 and F6 have potential antioxidant abilities. In the future, we can further investigate the potential effects on other functions, and the molecular and biochemical mechanisms of these biofunctional activities.

## 6. Patents

This research obtained a Taiwanese invention patent, patent cert. No.: I706728.

## Figures and Tables

**Table 1:** Chemical composition analysis of FB, F3, and F6.

Sample	Soluble Protein (mg/mL)	Peptides (mg/mL)	Total Phenolic (μg/mL)
FB	10.06 ± 0.53 ^c^	4.42 ± 0.22 ^b^	0.71 ± 0.02 ^c^
F3	19.33 ± 0.81 ^b^	10.43 ± 1.85 ^a^	3.03 ± 0.06 ^a^
F6	21.14 ± 0.77 ^a^	11.89 ± 0.45 ^a^	2.36 ± 0.02 ^b^

Data are expressed as mean ± SD (*n* = 3). Duncan’s multiple range tests were performed, with different superscripts in a column (a,b,c) indicating significant difference (*p* < 0.05). FB: fish bone; F3: fish bone fermented with *Monascus purpureus* for 3 days; F6: fish bone fermented with *Monascus purpureus* for 6 days.

**Table 2:** Free amino acid composition analysis (mg/100 mL) of FB, F3, and F6.

Parameter		FB	F3	F6
Essential amino acid	Histidine	10.10 ± 0.3 ^b^	25.76 ± 1.5 ^a^	23.63 ± 2.6 ^a^
	Isoleucine	0.43 ± 0.0 ^c^	3.11 ± 0.1 ^a^	0.72 ± 0.0 ^b^
	Leucine	0.96 ± 0.0 ^b^	6.47 ± 0.3 ^a^	1.10 ± 0.1 ^b^
	Lysine	2.82 ± 0.2 ^a^	0.21 ± 0.0 ^b^	3.05 ± 0.2 ^a^
	Methionine	0.68 ± 0.0 ^c^	5.62 ± 0.2 ^a^	2.68 ± 0.3 ^b^
	Phenylalanine	1.35 ± 0.1 ^b^	1.10 ± 0.0 ^c^	2.18 ± 0.1 ^a^
	Threonine	0.93 ± 0.0 ^c^	1.44 ± 0.0 ^b^	1.80 ± 0.2 ^a^
	Tryptophan	0.11 ± 0.0 ^b^	0.20 ± 0.0 ^b^	1.03 ± 0.1 ^a^
	Valine	0.88 ± 0.1 ^b^	5.82 ± 0.1 ^a^	0.46 ± 0.0 ^c^
	Total	18.26 ± 3.1 ^c^	49.74 ± 8.0 ^a^	36.64 ± 7.4 ^b^
Non-essential amino acid	Alanine	1.87 ± 0.0 ^c^	11.07 ± 0.1 ^a^	2.15 ± 0.1 ^b^
	Arginine	0.64 ± 0.0 ^b^	0.44 ± 0.0 ^c^	1.47 ± 0.2 ^a^
	Asparagine	0.04 ± 0.0 ^c^	0.95 ± 0.0 ^b^	1.76 ± 0.0 ^a^
	Aspartic acid	0.06 ± 0.0 ^c^	1.16 ± 0.1 ^b^	1.69 ± 0.1 ^a^
	Cystine	0.13 ± 0.0 ^b^	0.18 ± 0.0 ^a^	0.14 ± 0.0 ^a b^
	Glutamic acid	1.80 ± 0.1 ^c^	5.73 ± 0.3 ^a^	3.28 ± 0.1 ^b^
	Glutamine	0.03 ± 0.0 ^c^	0.21 ± 0.0 ^b^	0.27 ± 0.0 ^a^
	Glycine	2.33 ± 0.0 ^b^	15.32 ± 2.2 ^a^	2.55 ± 0.0 ^b^
	Proline	0.55 ± 0.1 ^c^	1.19 ± 0.1 ^b^	1.30 ± 0.0 ^a^
	Serine	0.26 ± 0.0 ^c^	2.29 ± 0.1 ^a^	1.82 ± 0.1 ^b^
	Tyrosine	0.79 ± 0.0 ^b^	0.14 ± 0.0 ^c^	1.60 ± 0.0 ^a^
	Total	8.51 ± 0.8 ^c^	38.70 ± 5.1 ^a^	18.02 ± 0.9 ^b^
Total free amino acids		26.76 ± 2.2 ^c^	88.44 ± 6.5 ^a^	54.66 ± 5.0 ^b^

Data are expressed as mean ± SD (*n* = 3). Duncan’s multiple range tests were performed, with the same superscripts (a,b,c) assigned to an amino acid indicating a non-significant difference (*p* < 0.05). FB: fish bone; F3: fish bone fermented with *Monascus purpureus* for 3 days; F6: fish bone fermented with *Monascus purpureus* for 6 days.

## Data Availability

Not applicable.

## Sample Availability

Samples of the compounds are not available from the authors.
